# Simultaneous control of three degrees of freedom in perfect vector vortex beams based on metasurfaces

**DOI:** 10.1515/nanoph-2024-0709

**Published:** 2025-02-07

**Authors:** Siyang Li, Yaqin Zheng, Changda Zhou, Guoli He, Zhonghong Shi, Haoyang Li, Zhang-Kai Zhou

**Affiliations:** State Key Laboratory of Optoelectronic Materials and Technologies, School of Physics, 26469 Sun Yat-Sen University, Guangzhou 510275, China

**Keywords:** perfect vector vortex beams, metasurfaces, polarization distribution, beam shaping

## Abstract

The perfect vector vortex beams (PVVBs) have played an important role in various fields due to their advantages of unique vortex features, flexible polarization distribution and multiple degrees of freedom (DoFs). The simultaneous and precise control over multiple DoFs, such as the polarization distribution, beam shape and position which greatly influence various characteristics of PVVBs, holds paramount importance. However, it is still difficult to manipulate various DoFs in a multiplexing way and the control precision of polarization distribution only reaches the half-integer level, notably hindering the further application and development of PVVBs. Here, an approach that integrates holographic technique with geometric phase metasurfaces, experimentally demonstrates the multiplexing control of PVVBs over three DoFs, i.e., enabling the independent manipulation of non-uniform polarization distributions, beam shapes and spatial positions. Furthermore, non-integer polarization order of the generated PVVBs can be arbitrary non-integer numbers with a high resolution of 0.1, largely improving the control precision. With such multiplexing manipulation of PVVBs with high precision, we can provide abundant processing dimensions for information science and technologies, exhibiting broad application potentials in fields such as information encryption, high-speed optical communication, and precise particle manipulation.

## 1 Introduction

The last decade has witnessed large amounts of research efforts devoted to the study of on-demand controlling perfect vector vortex beams (PVVBs) based on metasurface [1], [2], [3], [4], [5], [6], shedding light on various fields including optical chips [7], [8], [9], [10], quantum communication [11], microfabrication [12], microscopic imaging [13] and information coding [14], [15], [16], [17]. This trend can be attributed to two probable facts. On the one hand, the PVVB itself can sustain much more optical degrees of freedom (DoFs) for information technologies (such as information communication, processing, etc.) as compared with the scalar light field [18], [19], since its polarization characteristics vary spatially with a spiral phase wavefront carrying orbital angular momentum (OAM) [20]. On the other hand, with the employment of metasurface for manipulating PVVBs, the advantages of PVVBs can be inherited and applied by the on-chip devices with subwavelength scale, greatly promoting the miniaturization and integration of nanophotonic applications [21], [22], [23] with unprecedented features of high speed, large capacity, etc. [24].

Generally, there are three important DoFs that influence the generation and manipulation of PVVBs, as well as their corresponding applications. These three special DoFs are the polarization distribution, beam shape and appearance position. To be specific, the vector merit of light fields is described by their polarization distributions [25], and its order is defined as *m*, which indicates the number of polarization orientation cycles around the polarization singularity. Generally, when *m* is an integer, the linear polarization orientation on the beams changes uniformly by an integer multiple of 2*π*; whereas, when *m* is a fraction, the change corresponds to a fractional multiple of 2*π*. Notably, when a single value of *m* is maintained throughout the optical field, the rate of polarization azimuthal variation remains constant. By assigning distinct values of *m* to different regions within the optical field, it becomes feasible to achieve non-uniform polarization variations, thereby generating PVVB with characteristics of tailored distribution [2], [3], [26], [27], [28], [29]. Different from the PVVBs of integer order with uniformly varied distributions of polarization, the PVVBs with non-uniform polarization variations can display on-demand polarization states at desired areas, greatly improving the control precision of optical fields. These non-uniform polarization fields enable smaller focus areas compared to uniform ones [30], benefiting high-precision instruments and quantum information processing [31]. Though current methods such as noncanonical optical vortices [32] have been proposed, they essentially rely on amplitude modulation. However, generating PVVBs with non-uniform polarization distributions and distinguishable polarization order at the decimal level remains an underexplored area.

In addition, the shape and position of PVVBs are also important DoFs [4], [33], [34]. On the one hand, the manipulation of beam shape can control the gradient of the optical field, and thereby control the optical force caused by PVVBs. On the other hand, adjusting the position of PVVBs can enable precise steering and distribution of the optical field, providing a controllable DoF in a programmable and dynamic manner for optical information. Furthermore, it is recently demonstrated that the control of intensity distribution can be multiplexed with other DoFs [35], [36], [37], such as preserving polarization state information or OAM [38], [39], [40], [41], which offers novel avenues for high-capacity data transmission and optical encryption [42]. Therefore, to realize the on-demand modulating of PVVB shape and position, especially their multiplexing control with other DoFs, has become an important issue in the field of nanophotonics. However, in previous research, PVVBs were typically generated by the composite phase method, which combines the phase information of spiral phase plates, axicons and lenses [43], [44], [45]. Although these works can effectively control the topological charge and polarization modes of PVVBs at the semi-integer level, they are primarily applicable to vortex beams with complete annular intensity distributions, as axicon struggle with converting fractional-order and non-annular beams. Consequently, these approaches face challenges in generating fractional-order polarization modes, non-annular intensity distributions, and creating PVVBs at specific multiple positions. Moreover, few works have achieved multiplexing of the aforementioned three degrees of freedom of PVVBs.

In this letter, we propose and experimentally demonstrate a metasurface approach for simultaneously controlling those three DoFs mentioned above with high precision. By utilizing the holographic technique combined with the geometric phase control, we overlay two orthogonal circularly polarized perfect vortex beams (PVBs), achieving on-demand non-uniform polarization distribution at the decimal level, beam shape and position. The control precision of polarization orders has been greatly improved, with greater resolution from 0.5 to 0.1 at the decimal level. Besides, the distribution of linearly polarization states across the beam has become arbitrarily manipulable, while the shapes of the curves have evolved into more intricate forms. Based on this method, we can generate more complex and versatile beams, which will promote the integration of optical systems and equipment miniaturization, and have enormous application prospects that traditional optics do not have.

## 2 Results and conclusions

### 2.1 The principle of three-dimensional control of the perfect vector vortex beams

**Figure 1:**
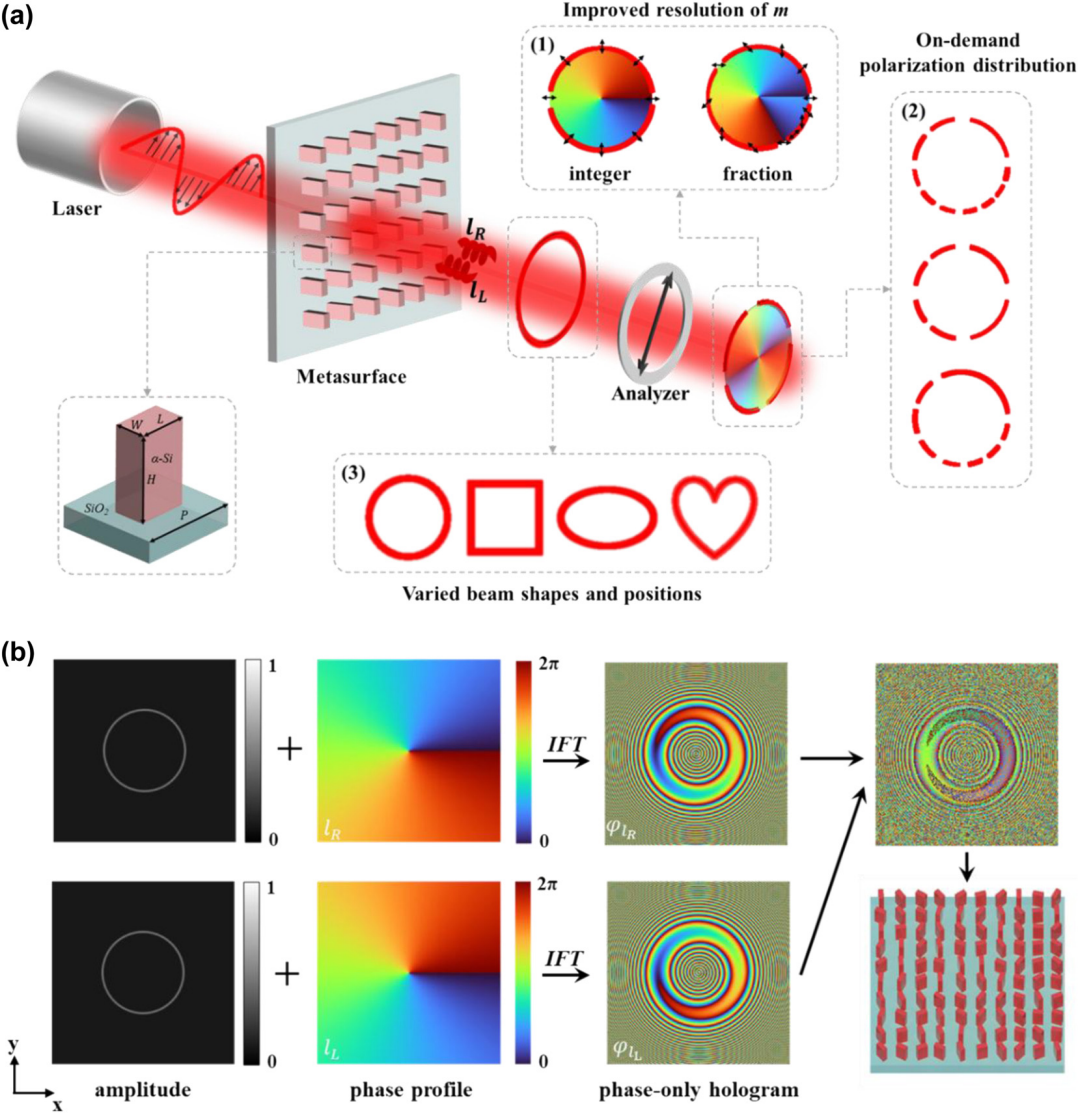
Schematic of the generation and design process for PVVBs with three degree of freedom control. (a) Schematic of the process for generating PVVBs using a metasurface. From left to right: laser, incident linearly polarized light, metasurface, output beam, linear polarizer, and the analyzed output light. The modulated output light is a superposition of PVBs with opposite topological charges. The superimposed electric field forms PVVBs with fractional polarization orders (1), on-demand polarization distribution (2), and varied beam shapes and positions (3). Black arrows denote polarization states. The bottom left shows a schematic of the unit cell of the metasurface. (b) Schematic of the design process for generating PVVBs. The first row shows the left-handed circularly polarized (LCP) component, and the second row shows the right-handed circularly polarized (RCP) component. The first column shows the target amplitude of the beam, the second column shows the target helical phase profile of the beam, and the third column shows the calculated phase information. The final phase information after superposing the two PVBs components is shown in the upper part of the fourth column, with the metasurface schematic below it.

Figure 1a illustrates the schematic of our metasurfaces for generating and manipulating PVVBs, which are arrays of amorphous silicon nanopillars on a glass substrate. With the height of the nanopillars fixed at 310 nm, a comprehensive sweep of the cross-sectional length and width of unit cells, is undertaken to compute the transmission efficiency and circular polarization conversion efficiency. After parameter selection to reach both high efficiencies, the transmission efficiency and polarization conversion efficiency reach the optimal balance point work in 633 nm wavelength LCP light incident when the length (*L*), width (*W*), height (*H*), and period (*P*) of the nanopillars are set as 175, 100, 310, 300 nm, respectively (detailed discussions are given in Figure S1). Based on the principle of Pancharatnam–Berry (PB) phase [46], [47], the nanopillars can induce distinct phase modulations to the left and right circularly polarized (LCP and RCP) lights. When the linearly polarized light with two orthogonal LCP and RCP components is incident upon the metasurfaces, the nanopillars can convert these components into orthogonal right-handed and left-handed perfect vortex beams (PVBs) with opposite phase distributions [48]. By superimposing these two orthogonally polarized PVBs, a PVVB with integer or fractional polarization orders can be formed on the observation plane, enhancing the resolution of *m*. Moreover, the capability to adjust the phase profile, amplitude and observation place of these orthogonally polarized PVBs allows for the superposition to generate PVVBs at specific positions with diverse non-uniform polarization distributions and varied shapes, such as circular, square, elliptical, heart-shaped, and other on-demand shapes.

To be specific, the generation idea of our method is to decompose the PVVBs with the desired polarization distribution, beam shape and position into a pair of orthogonally polarized PVBs (Figure 1b). For a PVVB with a radius *R*, the mathematical relation can be simply expressed by the following equation:

(1) $$E_{\text{PVVB}}\left(x,y\right)=E_{\text{PVB,R}}\left(x,y\right)+E_{\text{PVB,L}}\left(x,y\right)$$

where

(2) $$E_{\text{PVB,R}}\left(x,y\right)=\text{Aexp}\left[-\frac{{\left(\sqrt{x^{2}+y^{2}}-R\right)}^{2}}{\omega_{0}^{2}}\right]\exp\left(il_{R}\varphi \right)$$

and

(3) $$E_{\text{PVB,L}}\left(x,y\right)=Aexp\left[-\frac{{\left(\sqrt{x^{2}+y^{2}}-R\right)}^{2}}{\omega_{0}^{2}}\right]\exp\left(il_{L}\varphi \right)$$

where (*x, y*) is Cartesian coordinates, *φ* is the azimuthal angle whose values should range from 0 to 2π*, A* is the amplitude of the PVB (corresponding to the first column in Figure 1b), *ω*_0_ is the beam waist. *l*_L_ and *l*_R_ represent the topological charges of the PVBs with two orthogonal polarization states, respectively (*lφ* corresponds to the schematic in the second column of Figure 1b). The polarization order and topological charge of the generated PVVB can then be expressed as *m* = (*l*_R_ *− l*_L_) / 2 and *q* = (*l*_R_ *+ l*_L_) / 2, respectively. In our study, the configuration, *l*_R_ *= − l*_L_ is set and therefore one can derive that *m* = *l*_R_.

For a target optical field *E*(*x, y*), the complex amplitude of the optical field after the Fresnel diffraction transform can be simplified as:

(4) $$E_{d}\left(x,y\right)=E\left(x,y\right)\frac{\exp\left(jkz\right)}{j\lambda z}\exp\left[\frac{jk}{2z}\left(x^{2}+y^{2}\right)\right]$$

where *z* is the distance between the observation plane and the metasurface, and *k* is the wavenumber. In our method, the target optical fields are the two LCP and RCP PVBs which construct the required PVVB, and they should be generated by our metasurfaces based on holography. So, the initial phase information of the two orthogonal PVBs, i.e., the optical field phase presented by metasurfaces, should be derived through the inverse Fresnel diffraction transform (corresponding to the schematic in the third column of Figure 1b).

Furthermore, it can be observed that the amplitude and phase terms in Eqs. (2) and (3) are independent of each other. Hence, the phase term exp(*il*_R_ *φ*), exp(*il*_L_ *φ*) and amplitude term *A* can be configured separately. So, the exponential term in Eq. (2) can be replaced by:

(5) $$\exp\left(il_{R}\varphi \right)=\exp\left[i\overset{N}{\underset{n=1}{\sum }}\mathrm{rect}\left(\frac{N\varphi }{2\pi }-n+\frac{1}{2}\right)l_{Rn}\varphi \right]$$

where *N* and *l*_n_ represent the total number of sections and the topological charge of PVB in the *n*th section, respectively. The exponential term in Eq. (3) is replaced similarly. The number of *n* of the section increases counterclockwise from the positive *x*-axis of the Cartesian coordinate system.

Based on the above discussion, three conclusions can be made. Firstly, by assigning non-integer values to *m*, one can enable the creation of fractional polarization order. By rewriting the exponential term from axisymmetrical to non-axisymmetrical [26], polarization distribution with a non-uniform azimuthal variation rate can be achieved. This method involves dividing the 2*π* angular span into *N* sections, each assigned a unique phase variation as *l*_Rn_ *φ*, ultimately resulting in different polarization orders *m*_n_. Secondly, by defining the coordinates of each point on a two-dimensional curve in the *x − y* plane, the target optical field amplitude *A* can be set, thereby achieving a PVVB with a specific shape. Thirdly, by assigning two or several distances from sample to observation plane, multiple PVVBs can be integrated on a single metasurface. Here, *l*_*R*_ and *l*_*L*_ determine the helical phase distribution of the PVBs, *A* represents the amplitude distribution, and *z* denotes the diffraction distance. These parameters are mutually independent during holographic calculations, ensuring no conflicts when combining the three degrees of freedom. Compared with the majority of previous studies, our method extends and simultaneously controls previously rarely discussed degrees of freedom.

### 2.2 Improving the control precision of polarization order to the decimal level

For the PVVBs, the precise control of polarization distribution (i.e., the polarization order) is crucial for enhancing the ability to generate and manipulate optical fields. The polarization distribution control of PVVBs in current research based on the metasurface is at the semi-integer level, which means the generated PVVBs based on the current approaches can only have polarization order *m* as small as 1.5, and the minimal *m* resolution should also be 0.5. However, herein we will show the polarization distribution control at the decimal level, demonstrating the PVVBs with arbitrary value of *m*, as well as the improvement of *m* resolution from 0.5 to 0.1.

**Figure 2:**
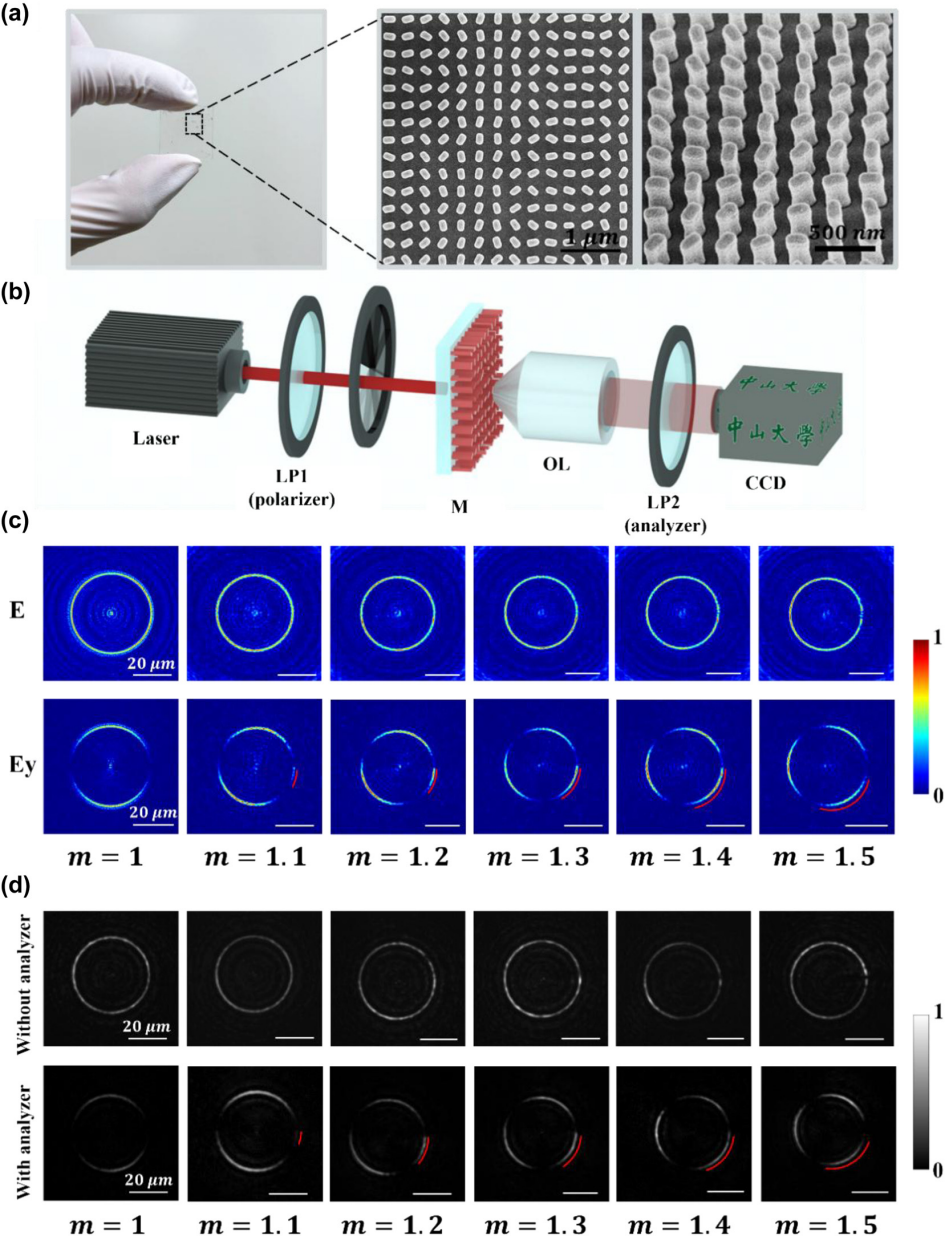
Fabrication and characterization of the metasurface and the results of PVVBs with fractional polarization orders. (a) Photo and SEM image of the fabricated metasurface with the top and tilted (30°) views, where black scale bars represent 1 μm and 500 nm, respectively. (b) Schematic of the experimental setup for characterizing the metasurface. (c) Simulation results of PVVBs with fractional polarization orders with white scale bar representing 20 μm. (d) Experimental results of PVVBs with fractional polarization orders with white scale bar representing 20 μm.

Figure 2 illustrates the generation and experimental demonstration approaches of our PVVB with the fractional polarization order. Based on the superposition of two PVBs with orthogonal phase wavefronts, a PVVB with vector properties is generated. The photos and typical scanning electron microscope (SEM) images of the fabricated metasurfaces are given in Figure 2a with the top and tilted (30°) views, showing the amorphous silicon nanopillars with varying rotation angles on a glass substrate. The sample size is 300 μm × 300 μm, with black scale bars representing 1 μm and 500 nm, respectively. The fabrication process of the sample is detailed in the Experimental Methods section, and Figure S1. Figure 2b presents the experimental setup for generating and measuring the PVVBs. The laser at 633 nm emitted by the source, after passing through the linear polarizer (LP1), becomes linearly polarized light and vertically illuminates the metasurface. After passing through the metasurface, the modulated light, which becomes a pair of orthogonally polarized PVBs with opposite helical phase wavefronts, generates the PVVBs with corresponding polarization orders. Finally, the output light is collected by an objective, a CCD camera, and an analyzer (i.e., a linear polarizer named LP2) which is inserted to analyze the polarization state of the output light, offering a straightforward approach for intuitively and easily obtaining the interpretable polarization distributions of outcomes.

The absolute value of the polarization order can be judged based on the relative length of the arcs after passing through an analyzer, as shown in Figure 2c and d, where the white scale bar represents 20 μm. It can be observed that when *m* = 1, only two arcs are present, signifying the typical PVVB with a *m* of 1. When a fractional number less than 1 is added to this topological charge, a third arc appears in the analyzed pattern. This third arc is notably shorter than the other two, with the two longer arcs representing the integer part of *m*, and the shorter arc representing the fractional part. The non-integer portion of *m* is denoted with a red arc, and as *m* changes from 1.1 to 1.5, the red arc correspondingly extends until at *m* = 1.5, all three arcs are of equal length. The experimental results, provided in Figure 2d, show a high degree of concordance with the simulation results (Figure 2c). It is demonstrated that the decimal-level precision in the polarization distribution of PVVBs has been achieved, which enhances the resolution and imaging capabilities [49] compared to integer-order PVVBs, thereby significantly improving the capacity and security of information transmission.

### 2.3 On-demand control of polarization distribution and beam shape

Besides the resolution of *m*, there are other three parameters that dominate the polarization distribution of PVVBs based on Eq. (5), namely the number of sections in the phase wavefront (*N*), and the topological charges carried by the PVB components in each section (*l*_Ln_ and *l*_Rn_, representing the *l*_n_ for LCP and RCP PVBs, respectively). For different phase wavefront sections of PVVB, the polarization order and topological charge can be calculated as *m*_n_ = (*l*_Rn_ – *l*_Ln_) / 2 and *q*_*n*_ = (*l*_Rn_ *+ l*_Ln_) / 2, respectively. Without loss of generality, we make each phase wavefront section have the same azimuthal angle and maintain the relationship *l*_Rn_ *=* −*l*_Ln_, leading to *m*_n_ = (*l*_Rn_ – *l*_Ln_) / 2 = *l*_Rn_. Under these conditions, the two parameters affecting the non-uniform polarization distribution of PVVBs are the angular extent *θ* = 2*π* / *N* and the polarization order *m*_n_. Therefore, as shown in Eq. (5), the arbitrary non-uniform polarization distribution can be obtained by assigning *m*_n_ and using a rectangular function to decide *N*.

**Figure 3:**
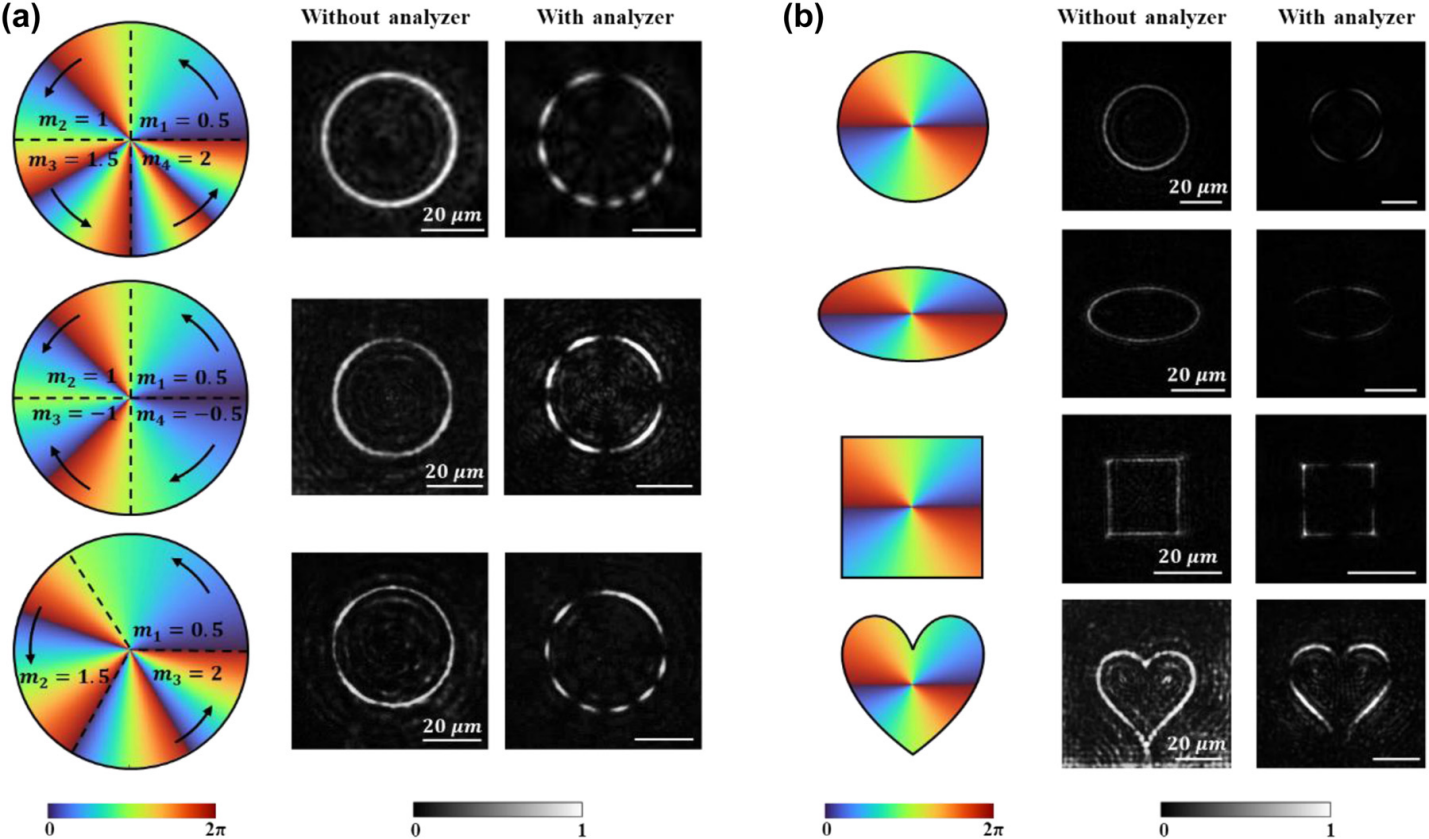
Experimental results of PVVBs with non-uniform polarization distributions and varied beam shapes. (a) Experimental results of PVVBs with different non-uniform polarization distributions with white scale bar representing 20 μm. The first column shows the schematic design of the polarization of PVVBs in each section, where the clockwise or counterclockwise black arrows represent the polarization order *m*_n_ of each section and the bottom of colored diagrams represents the phase wavefront. The second column shows the experimental results, and the third column shows the results after passing through an analyzer. The first row: *m*_1_ = 0.5, *m*_2_ = 1, *m*_3_ = 1.5, *m*_4_ = 2, the second row: *m*_1_ = 0.5, *m*_2_ = 1, *m*_3_ = −1, *m*_4_ = −0.5, the third row: *m*_1_ = 0.5, *m*_2_ = 1.5, *m*_3_ = 2. (b) Experimental results of PVVBs with different beam shapes with white scale bar representing 20 μm. From top to bottom, they are circle, ellipse, square, and heart-shaped. The first column shows the light intensity distribution curves and phase wavefront combination schematic, where the bottom of colored diagrams represents the phase wavefront. The second column shows the experimental results, and the third column shows the results after passing through an analyzer.

Figure 3a shows the experimental results of PVVBs with various non-uniform polarization distributions. In the first column of Figure 3a, the schematic diagrams of phase profile settings are drawn, in which the polarization order *m*_n_ of each section is labeled and indicated by a clockwise or counterclockwise black arrow. The colored phase wavefront diagram at the bottom of the diagrams represents *l*_Rn_ *φ*. For the parameter *m*_n_, its sign is the trend of linear polarization orientation variation in each section, while |2*m*_n_| represents the number of arcs observed after passing through an analyzer. The polarization distribution on PVVBs can be indirectly determined with the help of an analyzer as we have explained in Figure 2b, which can be compared to theoretical values of *m*_n_ for accuracy validation.

Experimental results are presented in the second and third columns (corresponding simulation results are provided in Figure S2). As seen in the first and second rows, the azimuthal angles of PVVBs are divided into four equal segments, i.e., *θ* = 90°. The distinction lies in the sign of *m*_n_: all positive in the former (i.e., the first row), but positive for *m*_1_ and *m*_2_ and negative for *m*_3_ and *m*_4_ in the latter (i.e., the second row). During the experiment, the polarization direction of incident light was maintained orthogonal to the analyzer’s direction, with the polarization orientations of the linear polarizers LP1 and LP2 (shown in Figure 2b) being set orthogonal to each other and rotated synchronously. As observed in Videos 1 and 2 in the Supplementary Materials, with the polarization state of incident light varying, the arcs within each section of the PVVBs are observed to exhibit a rotation pattern consistent with the theoretical polarization mode *m*_n_. This phenomenon indicates that the non-uniform polarization distributions across sections on the PVVBs are independent to each other. Furthermore, when the signs of polarization orders between the adjacent sections are opposite, it can be observed that the two arcs run in opposite directions or away from each other, verifying the consistency between the theoretical designs and experimental results.

The third row of PVVB features an azimuthal angle divided into three equal segments, i.e., *θ* = 120°. Although the experimental results show similarities with the light intensity distributions of the upper two rows, the difference lies in the division of the sector boundaries, where the arcs of each region disappear and reappear (Video 3 of the Supplementary Materials). In Figure S3, the relationship between light intensity and azimuthal angle across the cross-section of PVVBs, as derived from both simulations and experiments, is quantitatively compared and analyzed. It is observed that, regardless of whether from simulations or experiments, the azimuthal positions corresponding to the dark points within the beams are found to be almost identical to the theoretical design. In addition, the experimental results demonstrate that the polarization orders and azimuthal angle spans can be precisely designed by utilizing Eq. (5), enabling the modulation of non-uniform distributions of radially polarized light along curved paths within PVVBs, thereby providing enhanced performance [50] and flexibility in complex optical systems [51].

Apart from non-uniform polarization distributions, the beam shape [37] of PVVBs is also crucial, since it can directly impact the performance and application effectiveness of optical systems [33], [52]. For instance, in fields such as optical communication [53] and microscopic imaging, precise control over the beam shape has been shown to significantly enhance system resolution and efficiency. Then in the field of optical trapping [54] and micromanipulation, beam shaping enables the guidance of manipulated particles along specific energy flow directions [55]. However, achieving precise and arbitrary control of PVVB shape is challenging and seldom reported in metasurface systems.

Figure 3b shows the experimental results of four types of PVVBs with different beam shapes generated by our proposed method. In the first column, schematic diagrams are depicted for setting the intensity distribution curves (including circular, elliptical, square, and heart-shaped) and phase wavefronts. Following this, the corresponding experimental results are displayed in the second and third columns, where the intensity and polarization distributions of each PVVB are clearly shown. Notably, all types of beams exhibit uniform four-segment arcs after passing through an analyzer, validating the correctness of the set beam polarization mode *m* = 2. Corresponding simulation results are in Figure S4, where the simulation matches well with the experimental results. The experimental results demonstrate the capability of the adopted method, whereby, once the amplitude of the target optical field is expressed, the phase information of the metasurface, computed through inverse diffraction transformation, can generate PVVBs of arbitrary two-dimensional shapes.

### 2.4 Simultaneously controlling the three DoFs of the polarization distribution, beam shape and position

**Figure 4:**
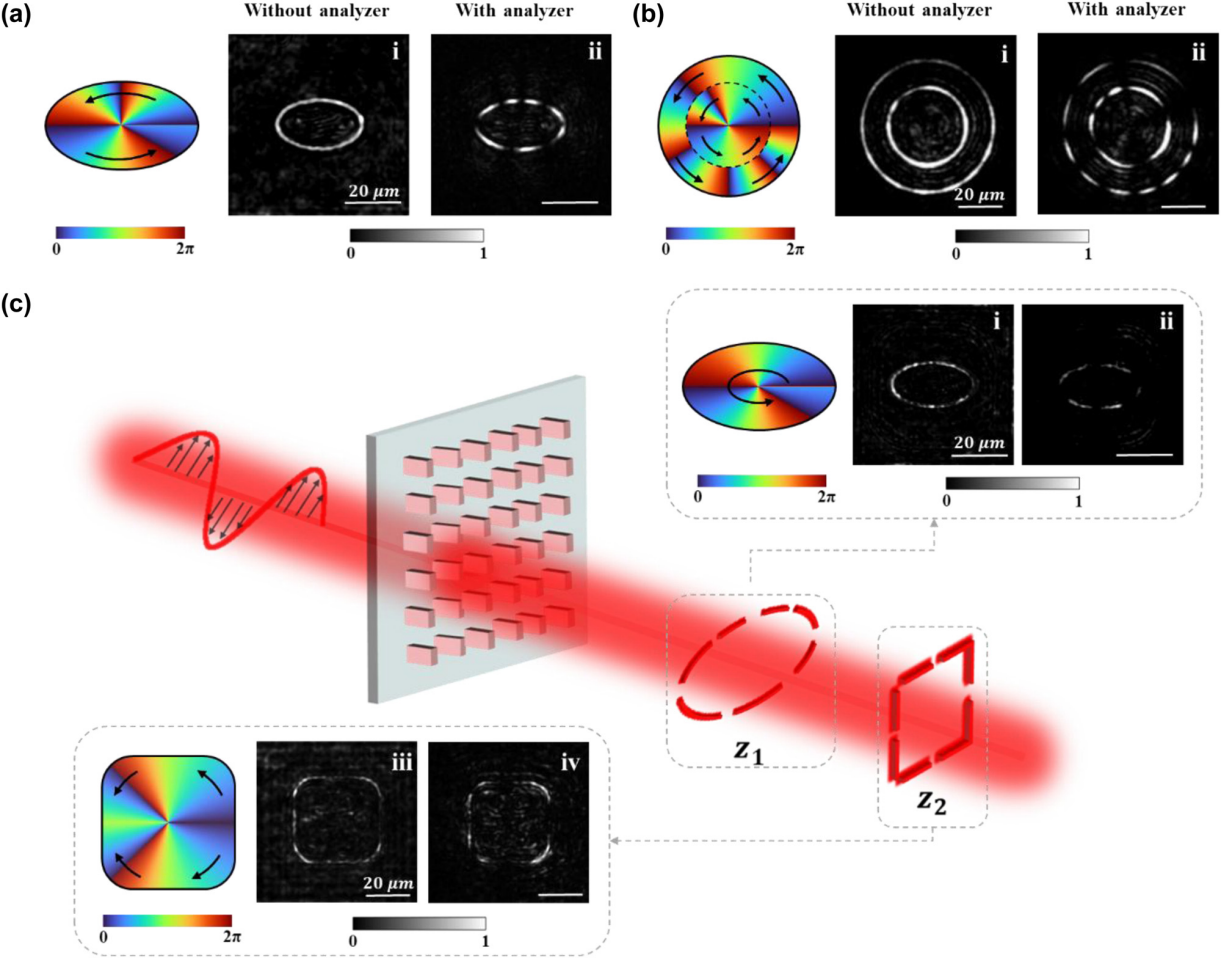
PVVBs with simultaneous control of multiple degrees of freedom. (a) The multiplexed PVVB where three degrees of freedom are controlled simultaneously. The polarization order of the upper half: *m*_1_ = 2, and the polarization order of the lower half: *m*_2_ = 1.2. (b) A hybrid PVVB composed of two concentric PVVBs with different non-uniform polarization distributions. The polarization orders of outer PVVB in each section: *m*_1_ = 0.5, *m*_2_ = 1, *m*_3_ = 1.5, *m*_4_ = 2, the polarization orders of inner PVVB in each section: *m*_1_ = 0.5, *m*_2_ = 1.5, *m*_3_ = 0.5, *m*_4_ = 0.5. (c) Schematic diagram of the PVVB of multiplexing three DoFs. PVVBs with fractional-order polarization modes, multiple non-uniform polarization distributions, and multiple intensity distributions are simultaneously realized at different distances from the observation plane. The polarization order of PVVB at plane *z*_1_: *m* = 2.2, the polarization orders of PVVB at plane *z*_2_: *m*_1_ = 0.5, *m*_2_ = 1, *m*_3_ = −1, *m*_4_ = −0.5. The first image of each numbered picture shows the schematic design of the polarization of PVVBs, where the clockwise or counterclockwise black arrows represent the polarization order *m* and the bottom of colored diagrams represents the phase wavefront. The white scale bar represents 20 μm.

In previous experimental results, we have demonstrated the independent manipulation of the polarization distribution and beam shape. Next, we will show that with the multiplexing strategy based on our method, it is possible to generate PVVBs and simultaneously control three DoFs, where the resolution of the polarization order can still be as high as 0.1. Figure 4 shows the multiplexed controlling of different DOFs, with the left and right figures presenting the experimental results of generated PVVBs with and without the polarization analyzer, respectively. In the first image of each numbered figure, the schematic diagrams of phase profile settings are drawn, in which the polarization order *m* is labeled and indicated by a clockwise or counterclockwise black arrow. The colored phase wavefront diagram represents *l*_Rn_ *φ* or *l*_R_ *φ*.

Firstly, we have achieved the multiplexing control of the polarization distribution and beam shape on a single PVVB (Figure 4a). Specifically, as depicted in Figure 4a, different from a circular annular shape, an elliptical PVVB featuring non-uniform polarization distribution has been successfully generated, with its azimuthal angle evenly divided into two regions, each spanning *θ* = 180°. In the upper half of the beam, a polarization order of *m*_1_ = 2 is imparted, resulting in four segment arcs as observed in the experimental results after passing through an analyzer. As previously mentioned, the polarization order indicates that the vibration azimuth of the linear polarization is rotated by a multiple of 2π radians. Therefore, the change in the vibration azimuth angle of linear polarization in each region can be determined, and then the corresponding polarization orders can be calculated to validate the design. The comparison between the arcs reveals that the azimuth swept by these four arcs is equal, indicating that the vibration azimuth angle of linear polarization has changed by a complete 4π, thus the polarization order of the upper half can be calculated as *m*_1_ = 4π/2π = 2. By the way, the uniform variation of linear polarization direction is projected onto the centrally symmetric circular beams, resulting in a uniform distribution of intensity after passing through an analyzer. However, the projected arc length appears uneven due to the elliptical intensity shape of this beam. Nevertheless, this irregularity does not impede the accurate reading of its polarization order during the calculation process. In contrast, the lower half of the beam adopts a polarization order of *m*_2_ = 1.2, resulting in arcs of varying lengths corresponding to the integer and fractional components. The proportional relationship between *φ*_1_ and *φ*_2_ is given by *φ*_2_/*φ*_1_ = 0.4, with the first two arcs sweeping by the azimuth angle of *φ*_1_, and the third arc sweeping *φ*_2_. Thus, the polarization order of the lower half is calculated as *m*_2_ = π × (2 + 0.4)/2π = 1.2. These results illustrate that our method experimentally enables the simultaneous control of the beam shape and polarization distribution with an *m* resolution of 0.1, which possesses the potential to enhance higher density of information encoding [56].

Additionally, we demonstrate the generation of multiple PVVBs by integrating two concentric annular PVVBs with different non-uniform polarization distributions (Figure 4b), which not only enriches the set of information dimensions but also significantly enhances the information processing capabilities of optical systems [57]. These superior characteristics suggest that hybrid PVVBs hold vast potential for applications in optical communication and storage. The azimuthal angles of both annular beams are equally divided into four sectors (*θ* = 90°), with the sign of *m*_n_ being positive. The results (Figure 4b ii) verify the consistency between the observed number of arcs in each section and the theoretical settings.

Subsequently, to further demonstrate the flexibility of our design approach, we proceed to demonstrate the generation of diverse PVVBs at different positions. As depicted in Figure 4c, two PVVBs with different shapes and varied non-uniform distributions of fractional polarization orders are generated at two predesignated observation planes: *z*_1_ = 30 μm and *z*_2_ = 100 μm, respectively, increasing the number of controllable DoFs from 2 to 3. At the plane *z*_1_ (Figure 4c i and ii), the PVVB exhibits a polarization mode of *m* = 2.2. At the plane *z*_2_ (Figure 4c iii and iv), the PVVB is segmented into regions, each of which has a specific polarization order: *m*_1_ = 0.5, *m*_2_ = 1, *m*_3_ = −1, *m*_4_ = −0.5. To reduce conflicts in generating two PVVBs simultaneously, two sets of nanopillar arrays were arranged in a randomly interleaved pattern. One array generates a PVVB with elliptical intensity distribution on the plane *z*_1_, while the other generates a PVVB with square intensity distribution on the plane *z*_2_. The arrangement ensures that the array primarily generates its intended elliptical PVVB on the plane *z*_1_, while the minor intensity response from the other array is uniformly dispersed as background light, minimizing interference with the elliptical PVVB. The validity of this design is corroborated by the arc-shaped annular distribution observed in the experimental results, thereby confirming the accuracy of our design scheme. By comparing the results in these two planes, the variations of the beam intensity and polarization distribution at different spatial locations can be clearly observed. The corresponding simulation results, provided in detail in Figure S5, exhibit a strong agreement with experimental findings, further reinforcing the reliability of our approach. To further improve imaging quality, the design and fabrication process could be optimized. Specifically, increasing the size of the metasurface, more light collected by objective lens can be manipulated, thereby enhancing intensity of signal and reducing noise. Additionally, refining the fabrication process can bring the geometric parameters of the nanopillars closer to their designed values, further improving the signal-to-noise ratio. The results presented in Figure 4 demonstrate that our design methodology enables the generation of PVVBs with diverse non-uniform polarization distributions, and beam shapes at different spatial positions, thereby facilitating flexible modulation of beam characteristics. These results underscore the versatility and precision of our method, providing a solid foundation for applications in sophisticated optical systems.

## 3 Conclusions

In this work, we systematically proposed and experimentally demonstrated a method based on all-dielectric metasurfaces for the multiplexing manipulation of PVVBs with high precision. By integrating holographic techniques with the geometric phase modulation of metasurfaces, two orthogonally circularly polarized PVBs are superposed, enabling the manipulation of non-uniform polarization distributions, beam shapes and generation positions of PVVBs. Due to the introduction of transverse and longitudinal control, numbers of PVVBs can be generated simultaneously at different longitude locations, providing a high freedom for position manipulation. Furthermore, for the control of polarization distribution, we have achieved the distinguishable polarization orders accurate to decimal, i.e., improving the order resolution from 0.5 to 0.1 which can obviously provide novel compact focusing field and photophysical properties. Our researches bring about an innovative approach for flexible and precise control of PVVBs, holding promising application potentials and values in frontier fields such as optical micromanipulation, high-density information encoding, and integrated photonic chip technologies.

## 4 Experimental methods

The designed metasurface is composed of amorphous silicon nanopillars on a glass substrate. First, the inductively coupled plasma enhanced chemical vapor deposition system is utilized to deposit a layer of amorphous silicon with a thickness of 310 nm on the fused silica substrate. Next, the negative resist, hydrogen silsesquioxane polymer (HSQ), is spin-coated on the top of the amorphous silicon layer through a glue slinger, and then the samples are baked on a hot plate with a temperature set at 90 °C for 3 min. A thin layer of aluminum is deposited on the top of the sample for conductivity by a vapor deposition device. After that, setting the acceleration voltage, beam current, and dose of the electron beam, the photoresist is patterned after exposure with the electron beam lithography (EBL). The sample is then immersed in tetramethyl-lammonium hydroxide (TMAH) for 2 min for development and the unexposed area was dissolved. Finally, the pattern is transferred onto the amorphous silicon layer with inductively coupled plasma reaction etching (ICP).

## Supplementary Material

Supplementary Material Details

Supplementary Material Details

Supplementary Material Details

Supplementary Material Details
