# Supplementary material for: Simultaneous control of three degrees of freedom in perfect vector vortex beams based on metasurfaces
Source: Nanophotonics. 2025 Feb 7;14(3):417–27. doi: 10.1515/nanoph-2024-0709 (PMC11831394; doi:10.1515/nanoph-2024-0709)
Supplement: Supplementary file 1 — Supplementary Material Details [file j_nanoph-2024-0709_suppl_001.docx]

*Supplementary Materials for*

**Simultaneous Control of Three Degrees of Freedom in Perfect Vector Vortex Beams Based on Metasurfaces**

*Siyang Li#, Yaqin Zheng#, Changda Zhou#, Guoli He, Zhonghong Shi, Haoyang Li, and Zhang-Kai Zhou**

State Key Laboratory of Optoelectronic Materials and Technologies, School of Physics, Sun Yat-Sen University, Guangzhou 510275, China

*Corresponding author e-mail: [*zhouzhk@mail.sysu.edu.cn*](mailto:zhouzhk@mail.sysu.edu.cn)

**1. The unit cell design of the metasurface**

**
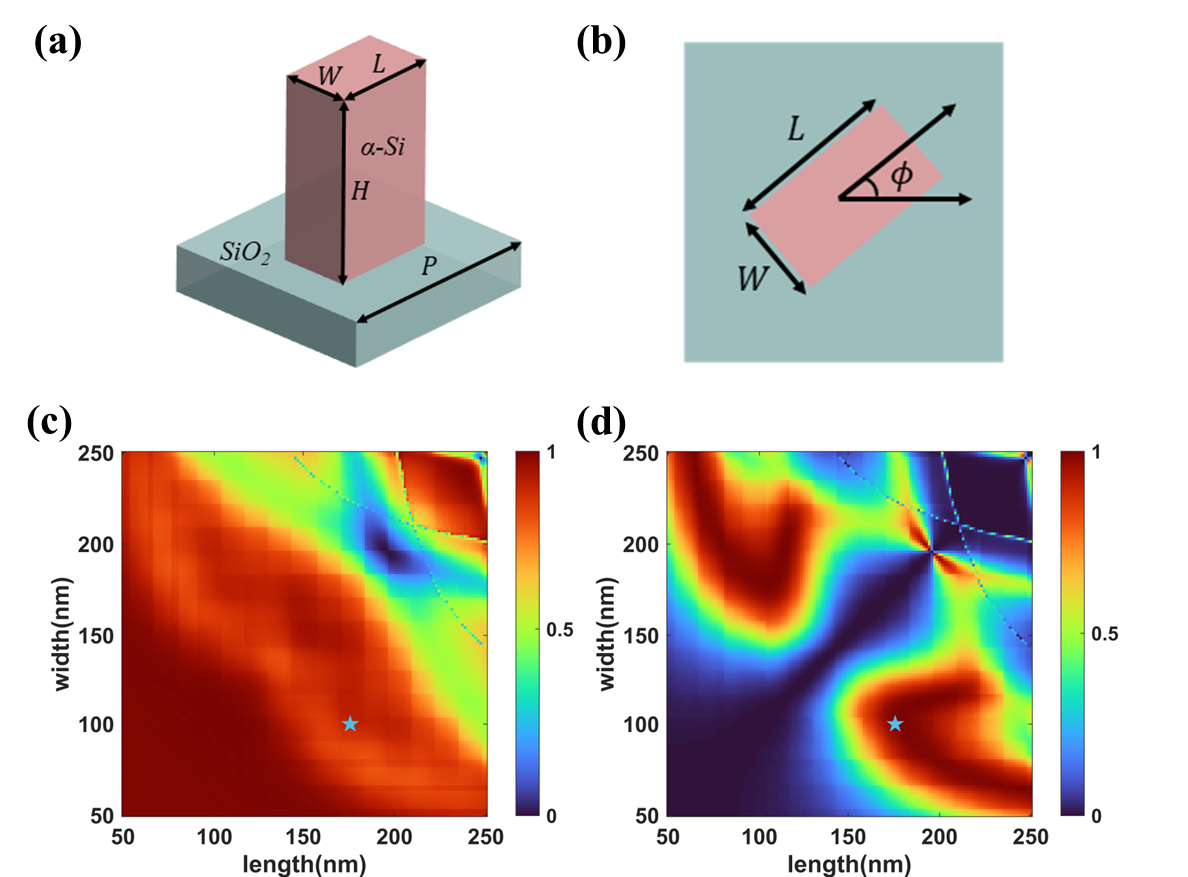
**

**Figure S1:** (a) Schematic of amorphous silicon nanopillar on a glass substrate. The length (*L*), width (*W*), height (*H*), and period (*P*) of the nanopillars are set as 175, 100, 310, and 300 nm. (b) Top view of the unit cell with a nanopillar rotated at angle *ϕ*. (c) Simulated transmission efficiency of nanopillars of different sizes. (d) Simulated polarization transformation efficiency of nanopillars of different sizes.

The unit cell design of the proposed metasurface comprises an array of spatially varying amorphous silicon nanopillars on a glass substrate, as depicted in Fig.S1(a). To evaluate the performance of these nanopillars with varying dimensions, finite-difference time-domain (FDTD) simulations were performed at a wavelength of 633 nm, maintaining a constant periodicity (P) of 300 nm between adjacent nanopillars. The simulation environment employed periodic boundary conditions along the x and y directions, while the perfectly matched layers (PML) boundary condition is utilized along the z direction to mimic an open boundary. The incident optical field is configured as a circularly polarized plane wave. With the height fixed at 310 nm, a comprehensive sweep of the cross-sectional length (ranging from 50 to 250nm) and width of unit cells, is undertaken to compute the transmission efficiency (Corresponding to Fig.S1(c)) and circular polarization conversion efficiency (Corresponding to Fig.S1(d)) for various sizes. Based on this analysis, an optimal unit cell size is identified, wherein both the transmission efficiency and polarization conversion efficiency attain high values when the length (*L*) of the nanopillar is 175 nm and the width (*W*) is 100 nm.

In our simulation study, the desired phase distribution can be achieved by controlling the rotation angles of the nanopillars. When the nanopillar rotates from 0° to 180°, the introduced phase exactly covers the range of 2π. The phase information of metasurface can be calculated using MATLAB software, leveraging the principle of Fresnel diffraction and the superposition of perfect vector beams (PVBs). The incident optical field is set as a plane wave with linear polarization state. To visualize the results of different electric field components, monitors were strategically positioned near the theoretical observation plane (z) within the simulation domain.

**2. Generation and manipulation of PVVBs with on-demand, non-uniform polarization distributions**

**
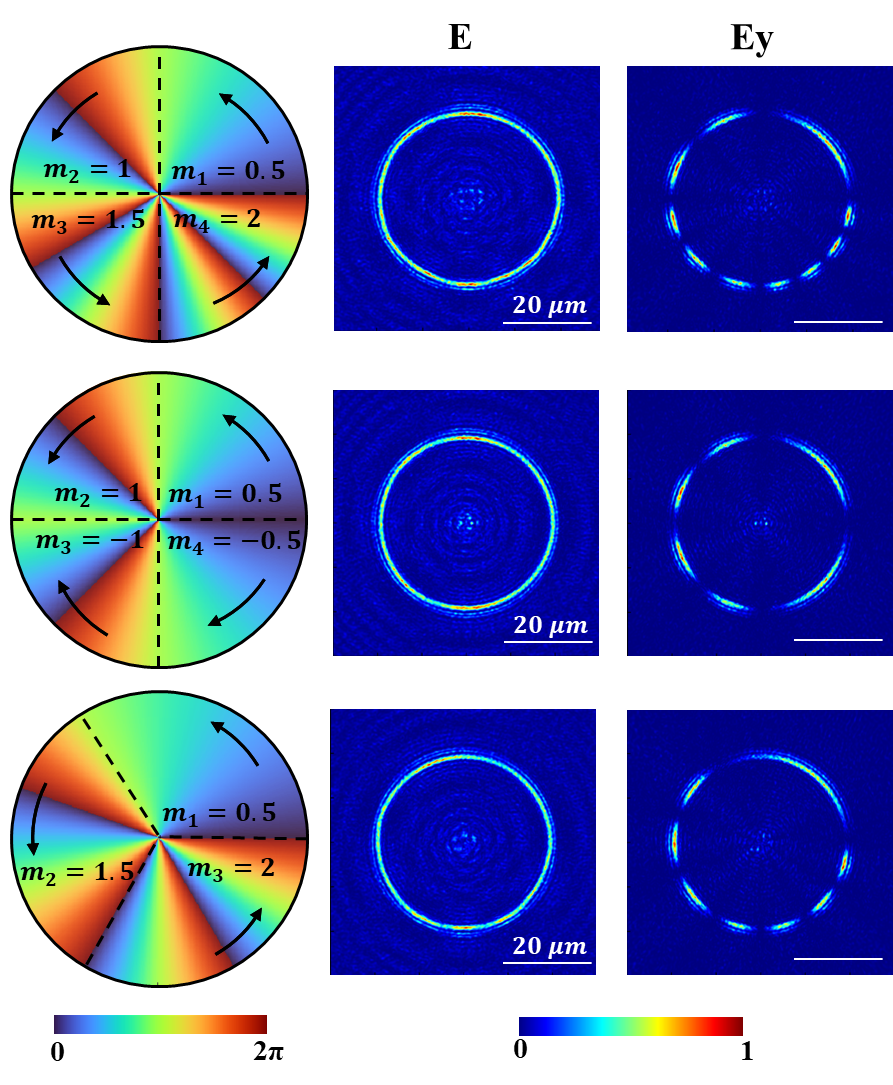
**

**Figure S2:** Simulation results of PVVBs with different non-uniform polarization distributions. The first column shows the schematic design of the polarization of PVVBs in each section, where the clockwise or counterclockwise black arrows represent the polarization order *m*_n_ of each section and the bottom of colored diagrams represents the phase wavefront. The second column shows the simulation results and the third column shows the results only containing the y-component of the electrical field, where white scale bar representing 20 μm. The polarization distribution of three PVVBs is designed as follows: the first row: *m*_1_ = 0.5, *m*_2_ = 1, *m*_3_ = 1.5, *m*_4_ = 2, the second row: *m*_1_ = 0.5, *m*_2_ = 1, *m*_3_ = -1, *m*_4_ = -0.5, the third row: *m*_1_ = 0.5, *m*_2_ = 1.5, *m*_3_ = 2.

**3. The quantitative relation between intensity distribution along azimuthal angle**

**
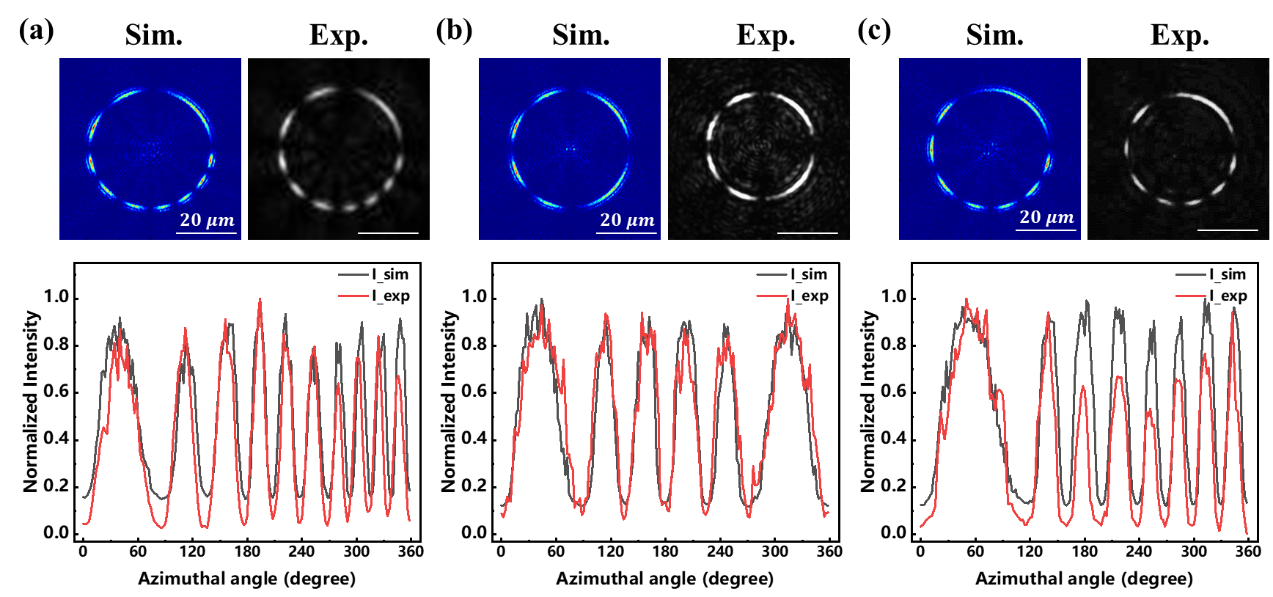
**

**Figure S3:** The relation between intensity distribution and azimuthal angle across the cross-section of beams. The first row in (a)-(c) represents simulation results only containing the y-component of the electrical field and experimental results after passing through an analyzer of three PVVBs, where white scale bar representing 20 μm. The second row is three sets of comparison curves for the intensity distribution along the azimuthal angle. The simulation and experimental results are shown in black and red solid curves, respectively.

**4. Generation and manipulation of PVVBs with different beam shapes**

**
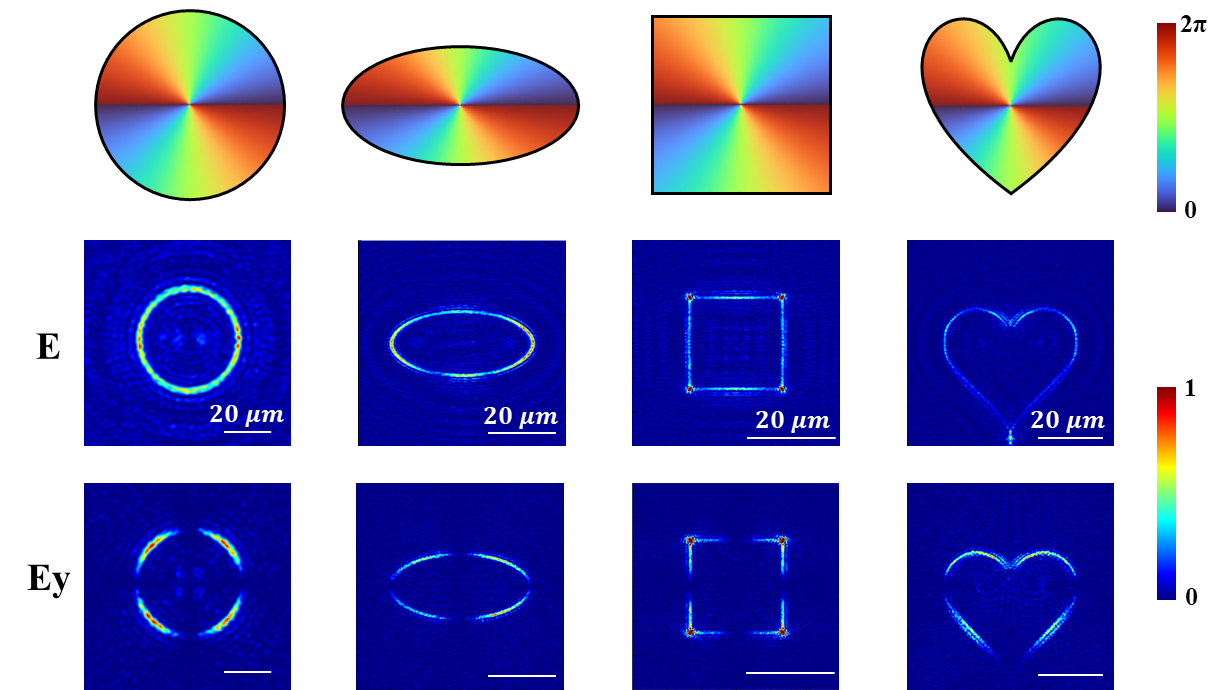
**

**Figure S4:** Simulation results of PVVBs with different beam shapes. From left to right, they are circle, ellipse, square, and heart-shaped. The first row shows the light intensity distribution curves and phase wavefront combination schematic. The second row shows the simulation results, and the third row shows the results only containing the y-component of the electrical field, where white scale bar representing 20 μm. The polarization order of all four PVVBs is *m* = 2.

**5. Generation and simultaneous manipulation of PVVBs controlling the three DoFs of the polarization distribution, beam shape and position**

**
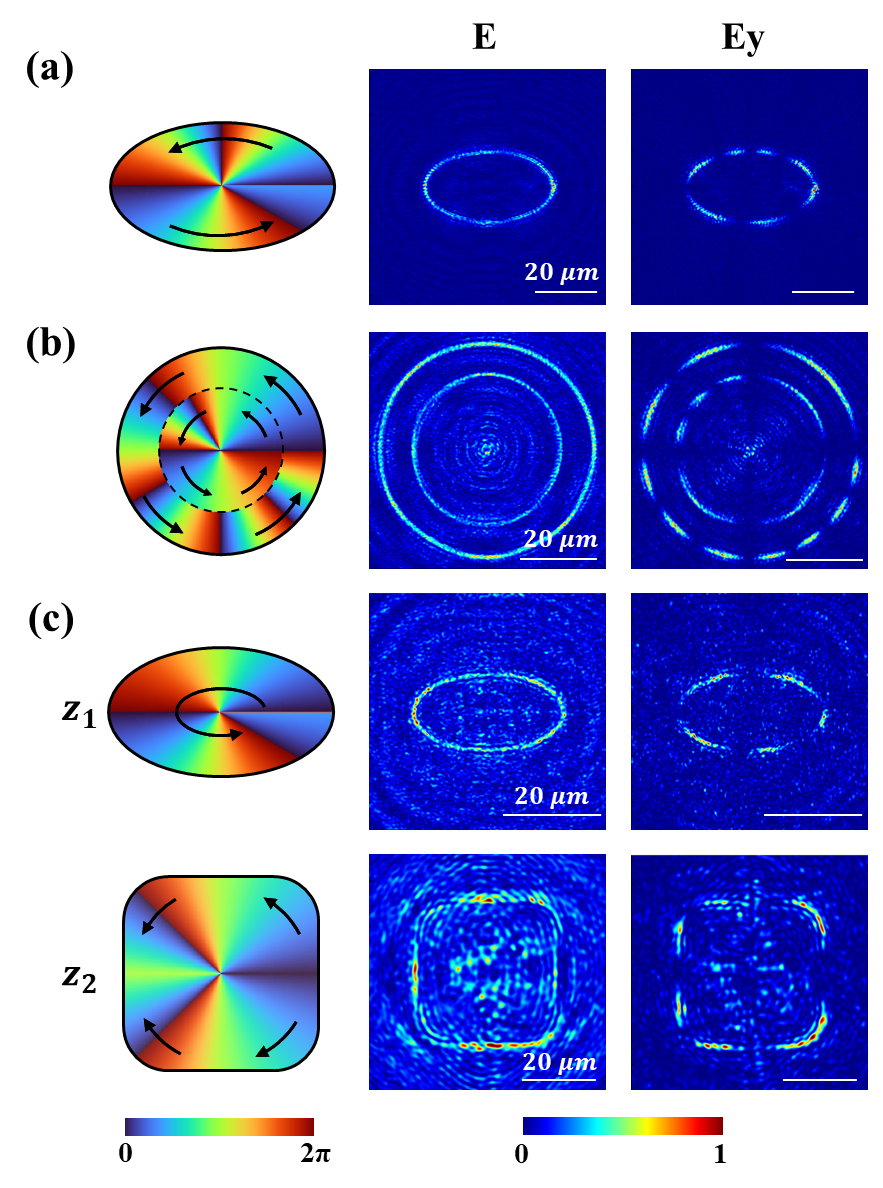
**

**Figure S5:** (a) The simulation result of multiplexed PVVB where three degrees of freedom are controlled simultaneously. The polarization order of the upper half: *m*_1_ = 2, and the polarization order of the lower half: *m*_2_ = 1.2. (b) The simulation result of the hybrid PVVB composed of two concentric PVVBs with different non-uniform polarization distributions. The polarization orders of outer PVVB in each section: *m*_1_ = 0.5, *m*_2_ = 1, *m*_3_ = 1.5, *m*_4_ = 2, the polarization orders of inner PVVB in each section: *m*_1_ = 0.5, *m*_2_ = 1.5, *m*_3_ = 0.5, *m*_4_ = 0.5. (c) The simulation result of the PVVB of multiplexing three DoFs. PVVBs with fractional-order polarization orders, multiple non-uniform polarization distributions, and multiple intensity distributions are simultaneously realized at different distances from the observation plane. The polarization order of PVVB at plane *z*_1_: *m* = 2.2, the polarization orders of PVVB at plane *z*_2_: *m*_1_ = 0.5, *m*_2_ = 1, *m*_3_ = -1, *m*_4_ = -0.5. The first image at each row shows the schematic design of the polarization of PVVB, where the clockwise or counterclockwise black arrows represent the polarization order *m* and the bottom of colored diagrams represents the phase wavefront. The white scale bar represents 20 μm.
